# Testing the associations between poverty stigma and mental health: The role of received stigma and perceived structural stigma

**DOI:** 10.1177/00207640241296055

**Published:** 2024-11-09

**Authors:** Greig Inglis, Edward Sosu, Fiona McHardy, Isabel Witteveen, Pamela Jenkins, Lee Knifton

**Affiliations:** 1 University of the West of Scotland, Paisley, UK; 2 University of Strathclyde, Glasgow, UK; 3 The Poverty Alliance, Glasgow, UK; 4 Mental Health Foundation, Glasgow, UK

**Keywords:** Poverty, stigma, mental health, well-being

## Abstract

**Background:**

Previous research has documented how people living on low incomes in the United Kingdom (UK) and internationally experience various forms of poverty stigma. The purpose of this study was to quantitatively examine how experiences of poverty stigma are associated with mental health outcomes.

**Methods:**

An online, cross-sectional survey was conducted with 1,000 adults living in predominantly low- and middle-income households in the UK. The survey included a questionnaire designed to measure participants’ experiences of different forms of poverty stigma, as well as measures of anxiety, depression and mental well-being.

**Findings:**

Exploratory and confirmatory factor analyses of the poverty stigma questionnaire supported a two-factor solution. One factor reflected participants’ experiences of being mistreated and judged unfairly by other people because they live on low income (received stigma) and the other factor reflected participants’ perceptions of how people living in poverty are treated by media outlets, public services and politicians (perceived structural stigma). Both received and perceived structural stigma were independently associated with anxiety, depression and mental well-being and these relationships persisted after controlling for socioeconomic indicators. There was also evidence that received stigma and perceived structural stigma partially mediated the relationships between financial hardship and mental health outcomes.

**Discussion:**

Experiences of received and perceived structural poverty stigma are both associated with mental health and well-being. This suggests that addressing interpersonal and structural forms of poverty stigma may help to narrow socioeconomic inequalities in mental health.

## Introduction

Approximately 22% of the population in the UK (14.4 million people) were estimated to be living in poverty in 2021/22 (Joseph Rowntree Foundation, 2024). People living in poverty are disproportionately affected my mental ill-health and are more likely to experience common mental health problems including anxiety and depression (Ridley et al., 2020). Stigma towards people experiencing poverty represents a range of psychosocial stressors through which poverty may affect mental health (Inglis et al., 2019). Link and Phelan (2001) suggest that stigma occurs when individuals are labelled as possessing socially salient characteristics and are associated with negative stereotypes. As a result, labelled persons are treated as a distinct social group (‘them’) that is separate from non-labelled persons (‘us’). Stigmatised individuals are consequently devalued and discriminated against, which leads to a range of social, economic and health inequalities (Hatzenbuehler et al., 2013; Link & Phelan, 2001).

The processes described by Link and Phelan (2001) give rise to several manifestations of stigma. Stigmatised persons may encounter discrimination from others (received stigma), believe that most people in society hold a negative view of people like themselves (perceived stigma) or expect that others will discriminate against them in the future (anticipated stigma). Stigma can also occur outside of interpersonal interactions, such as when discrimination is the result of institutional policies or practices (structural stigma; Pescosolido & Martin, 2015).

We use the term ‘poverty stigma’ to describe the collective forms of stigma that are experienced by people living on low incomes, which operate at institutional, interpersonal and intrapersonal levels (Inglis et al., 2019). At an institutional level, individuals may encounter unfair treatment when they try to access, for example, social security benefits, whilst the media can be a source of negative stereotypes about low-income communities. At an interpersonal level, people living in poverty may encounter discrimination from others in their community because of their financial situation, or they may be concerned about being treated unfairly by others in the future. In addition, self-stigma occurs when people living in poverty internalise negative stereotypes (Inglis et al., 2023).

Stigma is an important social determinant of mental health (Mak et al., 2007; Schmitt et al., 2014), and experiences of poverty stigma are associated with higher levels of negative mood (Chan et al., 2022) and depression (Mickelson & Williams, 2008; Turan et al., 2023), as well as lower self-esteem (Simons et al., 2017). Additionally, Hirsch et al. (2019) found that poverty stigma was negatively associated with a composite measure of mental health consisting of indicators such as emotional well-being and social functioning.

There is also evidence that discrimination mediates the relationship between financial hardship and health outcomes. For example, general measures of perceived discrimination (not attributed to any specific characteristic) have been found to mediate the relationship between socioeconomic disadvantage and self-reported health (Fuller-Rowell et al., 2018), as well as the relationship between poverty and allostatic load (Fuller-Rowell et al., 2012). Link et al. (2024) further report that experiences of discrimination and internalised feelings of shame relating to individuals’ level of education or financial situation mediate the relationship between socioeconomic position and several health outcomes.

There is growing interest in how experiences of poverty stigma are related to mental health, although few studies have explicitly tested whether experiences of poverty stigma mediate the relationship between financial hardship and mental health outcomes. In addition, the existing literature has focussed primarily on received stigma and self-stigma (Hirsch et al., 2019; Mickelson & Williams, 2008; Simons et al., 2017), while comparatively less attention has been given to other forms of poverty stigma such as perceived structural stigma. This is an important limitation, as qualitative studies have highlighted how people living in poverty experience these other types of poverty stigma (Inglis et al., 2019).

The purpose of the current study was therefore to test how experiences of poverty stigma are associated with mental health outcomes. We predicted that poverty stigma would be positively associated with mental ill-health and negatively associated with well-being. In addition, we expected that poverty stigma would mediate the association between financial hardship and mental health outcomes.

## Method

### Participants

The study was approved by the School of Education and Social Sciences Ethics Committee at the University of the West of Scotland (reference: 2023-21097-16521).

Our sample was predominantly low- and middle-income UK residents who completed an online cross-sectional survey. Participants were recruited through Prolific (www.prolific.com), a crowdworking platform where individuals can take part in paid research studies. Most users join the platform through word of mouth (Prolific, n.d.), and their primary motivation is to supplement their income (Berg et al., 2018).

Prolific has been found to produce high-quality data that is more reliable when compared to data collected through other crowdworking platforms (Douglas et al., 2023). Another advantage of Prolific is that users provide demographic information on their profiles, which allows researchers to pre-screen participants for studies (Palan & Schitter, 2018). Given our intention to recruit participants from low- and middle-income households, we used the pre-screening options to restrict the survey to Prolific users who lived in the UK and had a household income of less than £30,000. For context, the median household income in the UK is approximately £35,000 (Office for National Statistics, 2024).

The data were collected in September 2023 and participants were paid £1.85 for completing the survey.

The sample size was determined by considering the number of participants that would be required to develop a new measure of poverty stigma, and we followed White’s (2022) recommendation that between 750 and 1,000 participants are required for validation studies with both exploratory and confirmatory factor analyses. A total of 1,000 participants completed the survey. Of this, a small number of participants (*n* = 38) were excluded from our analysis due to having missing values on at least one variable (*n* = 35) or responding incorrectly to two attention check items that were embedded in the survey (*n* = 3). The final sample therefore consisted of 962 participants.

The mean age of participants was 42.14 years (*SD* = 14.44). Further demographic details for the sample are provided in Table 1.

**Table 1.** Participants’ demographic information.

Sociodemographic category	Frequency (%)
Country	
England	812 (84.4)
Scotland	88 (9.1)
Wales	53 (5.5)
Northern Ireland	9 (1)
Gender	
Male	404 (42)
Female	546 (56.8)
Other gender identity	12 (1.2)
Ethnicity	
White	857 (89.1)
Asian	43 (4.5)
Black	30 (3.1)
Mixed	23 (2.4)
Other ethnic group	9 (0.9)
Highest level of education	
Primary or secondary school	268 (27.9)
College or undergraduate university	564 (58.6)
Post-graduate degree	130 (13.5)
Employment	
Employed full-time	382 (39.7)
Employed part-time	220 (22.9)
Out of work and looking for a job	50 (5.2)
Out of work due to long-term sickness or disability	70 (7.3)
In education	39 (4.1)
Looking after home or family	84 (8.7)
Retired	76 (7.9)
Other employment status	41 (4.3)
Subjective poverty – financially just about getting by or worse	
No	281 (29.2)
Yes	681 (70.8)
Currently receive means tested benefits	
No	617 (64.1)
Yes	345 (35.9)
Used money or debt advice in last 12 months	
No	846 (87.9)
Yes	116 (12.1)
Used foodbank in last 12 months	
No	879 (91.4)
Yes	83 (8.6)
Food security	
High	481 (50)
Low	210 (21.8)
Very low	271 (28.2)
Unadjusted household income	
Less than £10,000	88 (9.1)
£10,000–£15,999	164 (17)
£16,000–£19,999	113 (11.7)
£20,000–£29,999	377 (39.2)
£30,000–£39,999	124 (12.9)
£40,000–£49,999	45 (4.7)
£50,000 or above	51 (5.3)
Equivalised household income	
Less than £10,000	303 (31.5)
£10,000–£19,999	415 (43.1)
£20,000–£29,999	183 (19)
£30,000 or above	61 (6.3)

Approximately 23% of the sample reported a household income of £30,000 or greater when completing the survey. This may be due to changes in some participants’ income or household since completing their Prolific profile, or it may reflect the unreliability of self-reported household income. We also calculated an equivalised household income figure for each participant, by taking the midpoint of each of the income bands and applying the modified OECD equivalence scale to adjust for the number of adults and children living in the household (Office for National Statistics, 2015). A total of 93.7% of participants reported an equivalised household income of less than £30,000.

### Measures

#### Experiences of poverty stigma

We sought to develop a new measure of poverty stigma for this study. To do so, we first identified the types of stigma that should be included in the scale by reviewing relevant qualitative research (e.g. Inglis et al., 2023) and the item content of existing scales measuring aspects of poverty stigma (e.g. Mickelson & Williams, 2008).

We identified four types of poverty stigma. The first was *received stigma*, which can be defined as a form of discrimination that is directed toward a person because they have a low income. Examples of received stigma include being disrespected or judged unfairly by others. The second was *anticipated stigma*, which we defined as an individual’s concerns that others will treat them unfairly because they have a low income. The third was *self-stigma*, which occurs when people on low incomes internalise negative stereotypes and apply these to themselves. This form of stigma also contains an affective component and may be experienced as feelings of shame. The final aspect was *perceived structural stigma* which relates to individuals’ beliefs about how people on low incomes are treated by institutions, public services or policy makers.

We drafted an initial measure with 32 items relating to these four types of poverty stigma. The measure was then piloted with four adults who had personal experience of poverty, who provided feedback on the wording of the items and response format. Each of these participants received a £20 shopping voucher for taking part in the research.

The measure was revised according to this feedback, and the final version consisted of 25 items: seven related to received stigma, seven related to anticipated stigma, five related to self-stigma and six related to perceived structural stigma. Participants were asked to rate each item based on their experiences over the past 12 months, using the following scale: (0) Never, (1) Rarely, (2) Sometimes, (3) Often, (4) Very often.

#### Financial hardship and socioeconomic status

Financial hardship was measured with the ‘shortage of money’ subscale from the Psychological Inventory of Financial Scarcity (Van Dijk et al., 2022). This three-item measure relates to whether individuals have enough money to pay their bills on time and buy the things that they need. Participants responded to each item on a seven-point scale ranging from Strongly Disagree to Strongly Agree. Cronbach’s α was .88.

Subjective poverty was measured with the item, ‘how well would you say you are managing financially these days?’, with the following response options: (1) living comfortably, (2) doing alright, (3) just about getting by, (4) finding it quite difficult and (5) finding it very difficult. Respondents who felt that they were just about getting by or worse were categorised as living in subjective poverty (Pearce, Lewis & Law, 2013).

Subjective social status was measured using the MacArthur ladder scale (Adler et al., 2000), where participants were asked to rank their socioeconomic position in relation to others in the UK on a ten-point scale.

Food insecurity was measured using the six-item form of the US Household Food Security Survey Module. Scores were categorised into groups of high, low and very low food insecurity following the guidance provided by the scale developers (US Department of Agriculture, n.d.).

Finally, participants were asked to report whether they were currently receiving any means-tested benefits, whether they had used a food bank or whether they had used any money or debt advice services in the past 12 months.

#### Mental health and well-being

Depression and anxiety were measured with the four-item Patient Health Questionnaire 4 (PHQ-4; Kroenke et al., 2009). Participants were asked to rate the extent to which they had experienced each item over the previous two weeks on the following scale: (0) not at all, (1) several days, (2) more than half the days and (3) nearly every day. Cronbach’s α was .93.

Mental well-being was measured using the seven-item Short Warwick-Edinburgh Mental Well-Being Scale (SWEMWBS; Stewart-Brown et al., 2009). Participants rated how frequently they had experienced various aspects of well-being over the previous two weeks using the following scale: (1) none of the time, (2) rarely, (3) some of the time, (4) often and (5) all of the time. Raw scores were converted to metric scores (Stewart-Brown et al., 2009) and Cronbach’s α was .88.

## Analytic approach

Our analytic approach involved exploratory and confirmatory factor analysis to examine the factor structure of the poverty stigma scale, as well regression analysis to examine associations between poverty stigma and mental health outcomes. To test the factor structure of the 25 poverty stigma items, the data set was randomly split in half and exploratory factor analysis (EFA) was undertaken on the first 481 responses and confirmatory factor analysis (CFA) on the second half.

For the EFA, the correlation matrix determinant was below 0.00001 which indicates multicollinearity (Field, 2018). To address this, pairs of variables that correlated ⩾.80 were identified, and one variable from each pair was removed. After doing so however, only two items relating to anticipated stigma and two items relating to self-stigma remained. We subsequently removed these items from the analysis, because at least three items are required to reliably identify a factor (Watkins, 2021). The final set of 10 items are provided in the Appendix. These items were then subjected to an EFA using principal axis factoring with oblique (promax) rotation because we expected that the extracted factors would be correlated. The Kaiser-Meyer-Olkin (KMO) measure of sampling adequacy was acceptable (.87). The KMO value of each individual item ranged between .80 and .96, exceeding the recommended minimum value of .50 (Field, 2018). Bartlett’s test of sphericity was also significant (χ^2^ = 3,199.56, *df* = 45, *p* < .001). The number of factors to retain was determined by the number of factors with eigenvalues greater than one and by checking the scree plot.

A CFA analysis was then conducted on the second half of the data to test the factor structure identified through the EFA. The CFA model was evaluated based on rules of thumb where the following values were taken to be indicative of acceptable model fit (Keith, 2015): Root Mean Square Error of Approximation (RMSEA) values of ⩽ .05; Tucker-Lewis Index (TLI) and Comparative Fit Index (CFI) values of ⩾.95; and Standardised Root Mean Square Residual (SRMR) values ⩽.08.

We used multiple regression to examine the association between poverty stigma and mental health outcomes, and parallel multiple mediation analyses were conducted to test whether poverty stigma mediated the association between financial hardship and mental health outcomes. The mediation analyses were conducted using Model 4 of PROCESS macro for SPSS (Hayes, 2022), and confidence intervals (CI) based on 50,000 bootstrap samples were calculated for each indirect effect.

## Results

### Dimensions of poverty stigma

Two factors were extracted for the EFA, and factor loadings for each item are displayed in Table 2.

**Table 2.** Factor loadings from an exploratory factor analysis of the 10 poverty stigma items.

Item	Factor 1	Factor 2
People have made negative assumptions about me because I don’t have much money.	**.90**	−.03
People have treated me badly because I don’t have much money.	**.86**	−.02
People have spoken down to me because I don’t have much money.	**.86**	.02
People have blamed me because I don’t have much money.	**.81**	−.02
Family or friends have excluded me from things because I don’t have much money.	**.60**	.05
People on low incomes are looked down on by television programmes in this country.	.06	**.84**
People on low incomes in this country receive a lower standard of public services.	−.11	**.82**
Public services in this country make you feel inadequate when you are living on a low income.	−.02	**.80**
Politicians in this country look down on people who live on low incomes.	.00	**.77**
People on low incomes are looked down on by newspapers in this country.	.11	**.73**

*Note*. Factor loadings of .60 or greater are displayed in bold.

Factor 1 explained 46.08% of the variance and was marked by high loadings of five items that relate to received poverty stigma. Factor 2 explained 18.87% of the variance and was marked by high loadings of five items that relate to perceived structural poverty stigma.

Confirmatory factor analysis was applied to the second half of the sample to further test this two-factor model, which indicated a poor fit overall (CFI = 0.88; TLI = 0.84; SRMR = 0.05; RMSEA = 0.16 (90% CI [0.14, 0.17]). The modification indices suggested that the model fit could be improved by covarying two pairs of items. One pair of items related to perceptions of how people living in poverty are represented by newspapers and television programmes, and another pair related to perceptions of how people living in poverty experience public services. This suggests that these pairs of items are associated with one another beyond what would be expected from the latent variable of perceived structural stigma. This is likely due to the similar meaning of the items, and so it seemed justifiable to apply these modifications (Sellbom & Tellegen, 2019). The modified model demonstrated a good fit (CFI = 0.99; TLI = 0.99; SRMR = 0.03; RMSEA = 0.05 (90% CI [0.03, 0.06]).

The internal consistency of both the received (Cronbach’s α = .90) and perceived structural stigma (Cronbach’s alpha = .89) scales was adequate. Bivariate and multivariate associations between both stigma scores and sociodemographic variables are presented in the Supplemental Tables 1 and 2.

### Associations between poverty stigma and mental health outcomes

The next set of analyses tested how received stigma and perceived structural stigma were associated with mental health outcomes. Descriptive statistics and bivariate correlations are presented in Table 3.

**Table 3.** Descriptive statistics and correlations between measures of poverty stigma and mental health.

Variable	1	2	3	*M* (*SD*)
1. Received stigma	1.00			4.36 (4.49)
2. Perceived structural stigma	.36			13.16 (4.66)
3. Anxiety and depression (PHQ-4)	.47	.38		4.83 (3.80)
4. Mental well-being (SWEMWBS)	−.33	−.30	−.70	19.74 (3.92)

*Note*. All correlations are statistically significant, *p* < .001.

Multiple regression analyses were conducted to further test the associations between poverty stigma and mental health outcomes. Two models were run for each outcome variable, where the first model only included poverty stigma scores as predictor variables and the second model included poverty stigma scores alongside various socioeconomic and demographic variables. The results from these multivariate models are reported in Table 4. Bivariate associations between sociodemographic variables and mental health outcomes are reported in Supplemental Table 3.

**Table 4.** Results of multiple regression analyses of the relationships between mental health outcomes, poverty stigma and sociodemographic variables.

	Anxiety and depression		Mental well-being	
Variable	*B* [95% CI]	*β*	*B* [95% CI]	*β*
Model 1				
Received stigma	0.33 [0.28, 0.38]***	.39	−0.22 [−0.28, −0.17]***	−.26
Perceived structural stigma	0.20 [0.15, 0.25]***	.24	−0.18 [−0.23, −0.13]***	−.21
Model 2				
Received stigma	0.18 [0.12, 0.23]***	.21	−0.07 [−0.13, −0.01]*	−.08
Perceived structural stigma	0.12 [0.08, 0.17]***	.15	−0.11 [−0.16, −0.06]***	−.13
Age	−0.04 [−0.05, −0.02]***	−.14	0.03 [0.01, 0.05]**	.11
Gender				
Male/other gender identity (reference)				
Female	0.44 [0.04, 0.84]*	.06	−0.12 [−0.57, 0.33]	−.02
Ethnicity				
White (reference)				
Other ethnicity	0.04 [−0.61, 0.68]	.00	−0.33 [−1.06, 0.39]	−.03
Subjective social status	−0.34 [−0.50, −0.18]***	−.13	0.34 [0.16, 0.52]***	.13
Financial hardship	0.17 [0.10, 0.23]***	.23	−0.15 [−0.22, −0.08]***	−.20
Subjective poverty				
No (reference)				
Yes	−0.26 [−0.83, 0.32]	−.03	−0.09 [−0.74, 0.56]	−.01
Income				
Below £10,000 (reference)				
£10,000–£15,999	0.31 [−0.48, 1.11]	.03	−0.55 [−1.45, 0.35]	−.05
£16,000–£19,999	0.23 [−0.65, 1.10]	.02	−0.49 [−1.47, 0.50]	−.04
£20,000–£29,000	0.31 [−0.47, 1.08]	.04	−0.03 [−0.90, 0.85]	.00
£30,000–£39,000	0.45 [−0.46, 1.37]	.04	−0.42 [−1.46, 0.61]	−.04
£40,000–£49,000	0.70 [−0.46, 1.86]	.04	−0.68 [−1.99, 0.63]	−.04
£50,000 or more	0.07 [−1.07, 1.20]	.00	−0.20 [−1.48, 1.08]	−.01
Education				
Primary or secondary school (reference)				
College or undergraduate degree	0.12 [−0.33, 0.57]	.02	0.34 [−0.18, 0.85]	.04
Postgraduate degree	0.71 [0.05, 1.37]*	.06	−0.36 [−1.11, 0.38]	−.03
Employment				
Employed full-time (reference)				
Employed part-time	0.28 [−0.26, 0.81]	.03	−0.03 [−0.63, 0.58]	.00
Out of work and looking for a job	−0.01 [−0.96, 0.94]	.00	−0.47 [−1.54, 0.61]	−.03
Out of work because of long-term sickness or disability	10.94 [1.06, 2.82]***	.13	−0.81 [−1.80, 0.18]	−.05
In education	0.06 [−0.98, 1.10]	.00	0.39 [−0.78, 1.57]	.02
Looking after home or family	−0.27 [−10.03, 0.49]	−.02	0.48 [−0.38, 1.34]	.03
Retired	0.67 [−0.21, 1.55]	.05	0.64 [−0.36, 1.63]	.04
Other employment status	0.38 [−0.63, 1.39]	.02	−0.46 [−1.60, 0.68]	−.02
Receives benefits				
No (reference)				
Yes	−0.49 [−0.95, −0.02]*	−.06	.62 [.09, 1.14]*	.08
Has used money/ debt advice in past 12 months				
No (reference)				
Yes	0.00 [−0.61, 0.62]	.00	0.52 [−0.17, 1.22]	.04
Has used foodbank in past 12 months				
No (reference)				
Yes	0.14 [−0.59, 0.87]	.01	0.03 [−0.79, 0.85]	.00
Food security				
High food security (reference)				
Low food security	0.02 [−0.55, 0.59]	.00	−0.10 [−0.74, 0.55]	−.01
Very low food security	0.93 [0.29, 1.57]**	.11	−1.04 [−1.76, −0.32]**	−.12

*Note. B* = unstandardised coefficient; CI = confidence interval; β = standardised coefficient.

* *p* < .05. **p < .01. ***p < .001.

For anxiety and depression, the first model was statistically significant (*R*^2^ = .28, *p* < .001), where both received (*B* = 0.33, β = .39, *p* < .001) and perceived structural stigma (*B* = 0.20, β = .24, *p* < .001) were positively associated with PHQ-4 scores. The second model, accounting for socioeconomic and demographic indicators, was also statistically significant (*R*^2^ = .40, *p* < .001), with both received (*B* = 0.18, β = .21, *p* < .001) and perceived structural stigma (*B* = 0.12, β = .15, *p* < .001) positively associated with anxiety and depression scores.

For mental well-being, the first model was statistically significant (*R*^2^ = .15, *p* < .001), with both received (*B* = −0.22, β = −.26, *p* < .001) and perceived structural stigma (*B* = −0.18, β = −.21, *p* < .001) negatively associated with mental well-being. The second model, controlling for socioeconomic and demographic indicators, was also statistically significant (*R*^2^ = 0.28, *p* < .001), with both received (*B* = −0.07, β *=* −.08, *p* = .016) and perceived structural stigma (*B* = −0.11, β = −.13, *p* < .001) being negatively associated with mental well-being .

Finally, we sought to test whether poverty stigma mediated the relationship between financial hardship and mental health outcomes. For each outcome variable, we initially examined the indirect effects of financial hardship through stigma without including any covariates, and then again whilst also adjusting for the full set of socioeconomic and demographic variables listed in Table 4. The results are presented below as unstandardised coefficients.

We first examined the indirect effect of financial hardship on anxiety and depression. In the unadjusted model, there was a statistically significant indirect effect of financial hardship through received stigma (*B* = 0.09, CI [0.068, 0.119]), as well as a statistically significant indirect effect of financial hardship through perceived structural stigma (*B* = 0.04, CI [0.030, 0.061]). In the adjusted model, controlling for socioeconomic and demographic variables, the indirect effect of financial hardship through received stigma remained statistically significant (*B* = 0.05, CI [0.027, 0.067]), although the indirect effect of hardship through perceived structural stigma was not statistically significant (*B* = 0.01, CI [0.000, 0.024]).

We next examined the indirect effect of financial hardship on mental well-being. In the unadjusted model, the indirect effects of financial hardship through both received stigma (*B* = −0.04, CIs [−0.073, −0.013]) and perceived structural stigma (*B* = −0.04, CIs [−0.056, −0.023]) were statistically significant. In the adjusted model, the indirect effect of financial hardship through received stigma was statistically significant (*B* = −0.02, CIs [−0.039, −0.001]) although as the upper level of the 95% confidence interval was so close to zero, this finding is likely to be sensitive to sampling error and should be treated with caution. The indirect effect of financial hardship through perceived structural stigma was not significant (*B* = −0.01, CIs [−0.023, 0.000])

## Discussion

The results of this study demonstrate that experiences of received and perceived structural poverty stigma are associated with mental health outcomes. In addition, received stigma partially mediated the relationship between financial hardship and mental health outcomes, even when controlling for sociodemographic variables. Perceived structural stigma also partially mediated the relationship between financial hardship and mental health outcomes in the unadjusted models, but these effects were attenuated and no longer statistically significant after controlling for sociodemographic variables.

Received stigma occurs when people living in poverty are rejected or devalued or by others because of their financial situation. The items used in the current study did not distinguish between specific sources of received stigma (besides one item which specifically mentioned ‘friends and family’), and so future research should explore the contexts in which received stigma occurs.

Perceived structural stigma refers to individuals’ perceptions of how people living in poverty are treated by institutions and decision makers. There are several reasons why perceived structural stigma may negatively affect mental health. For example, the perception that institutions and decision makers look down on people in poverty signals that those with low incomes are not valued in society, which is central to whether a person feels that they matter or not (Flett, 2022). Perceived structural stigma may also erode political and institutional trust, which are important aspects of social capital that predict well-being (Lindstrom & Mohseni, 2009).

The findings from this study support the view that addressing poverty stigma could be effective in reducing socioeconomic inequalities in mental health. This will likely require action on a range of targets across multiple social-ecological levels, including social policy, public attitudes and service delivery (Thornicroft et al., 2022).

At the social policy level for example, some authors have argued that socioeconomic disadvantage should be included in national equalities and anti-discrimination legal frameworks, which could be effective in challenging structural stigma (De Schutter, 2022).

At the public attitudes level, targeting negative stereotypes around poverty may be an effective means of reducing instances of received poverty stigma. There is some evidence that public attitudes relating to poverty change over time and may therefore be sensitive to intervention. For example, data from the British Social Attitudes survey show that negative attitudes toward benefits claimants in the UK rose sharply during the 1990s and 2000s, but that public opinion subsequently softened and became more generous during the 2010s (Geiger et al., 2023). This softening of attitudes occurred during a period of austerity and welfare reform, where the public became increasingly likely to recognise that levels of poverty are high in the UK. This also coincided with changes in media and political discourses around welfare, which became more generous and sympathetic from the early 2010s onwards. It is likely that each of these factors contributed to more generous attitudes toward benefit claimants (Geiger et al., 2023) and future research should explore how anti-stigma campaigns could further influence public opinion on these issues.

It is also important to reflect on how poverty stigma can be addressed within mental health services, given the intersections between poverty, poverty stigma and mental health outcomes. Several approaches that could be considered. For example, healthcare professionals’ biases towards and stereotyping of people from lower socioeconomic backgrounds have been found to adversely affect clinical decision making for disadvantaged patients (Job et al., 2024). Continuing staff training on issues relating to poverty may help to remediate these biases, as has been suggested by the American Psychological Association, which recognises that stigmatising attitudes held by mental health professionals can have a negative impact on the well-being of patients from low-income backgrounds (Juntunen et al., 2022).

Healthcare commissioners could also explore new methods of service delivery to support patients experiencing financial difficulties. For example, money and welfare advice services located within healthcare settings could be perceived as being more trustworthy and less stigmatising, which may in turn increase the uptake of these services leading to financial and health gains for patients (Reece et al., 2022).

Furthermore, practitioners working in social psychiatry are well placed to advocate for progressive social policies on the structural determinants of poverty and stigma, which could be effective in effecting change amongst policy makers (Knifton & Inglis, 2020). Mental health professionals should therefore be supported to recognise how poverty stigma operates alongside other social determinants of health to shape population mental health and health inequalities (Jeste & Pender, 2022).

Our findings have several limitations. First, the data were cross-sectional, and we cannot rule out the possibility of reverse causation, whereby poor mental health influences perceptions of poverty stigma. The data were also collected through non-probability online sampling which may limit the generalisability of the findings.

In addition, we had to omit several of the poverty stigma items from our analyses, which meant that we were unable to examine anticipated or self-stigma. Future research in this field should therefore continue to develop measures of poverty stigma that can comprehensively capture the variety of stigma processes that are reported by people with lived experience of poverty.

In sum, the results of this study demonstrate that both received and perceived structural stigma are associated with adverse mental health and well-being outcomes. The findings support the position that poverty stigma represents a range of psychosocial mechanisms through which poverty affects mental health, and addressing poverty stigma may therefore help to reduce socioeconomic inequalities in mental health.

## Supplemental Material

sj-docx-1-isp-10.1177_00207640241296055 – Supplemental material for Testing the associations between poverty stigma and mental health: The role of received stigma and perceived structural stigma Supplemental material, sj-docx-1-isp-10.1177_00207640241296055 for Testing the associations between poverty stigma and mental health: The role of received stigma and perceived structural stigma by Greig Inglis, Edward Sosu, Fiona McHardy, Isabel Witteveen, Pamela Jenkins and Lee Knifton in International Journal of Social Psychiatry

## Appendix: Poverty stigma scale

A list of statements is provided below, which may or may not apply to you. Please read each statement and then decide how much you agree or disagree with that statement, based on your experiences over the past 12 months.

1. People have made negative assumptions about me because I don’t have much money.
2. People have treated me badly because I don’t have much money.
3. People have spoken down to me because I don’t have much money.
4. People have blamed me because I don’t have much money.
5. Family or friends have excluded me from things because I don’t have much money.
6. People on low incomes are looked down on by television programmes in this country.
7. People on low incomes in this country receive a lower standard of public services.
8. Public services in this country make you feel inadequate when you are living on a low income.
9. Politicians in this country look down on people who live on low incomes.
10. People on low incomes are looked down on by newspapers in this country.

Each item is rated using the following scale:

0 – Never
1 – Rarely
2 – Sometimes
3 – Often
4 – Very often

Scoring:

Received stigma: 1 + 2 + 3 + 4 + 5

Perceived structural stigma: 6 + 7 + 8 + 9 + 10
