# Supplementary material for: Testing the associations between poverty stigma and mental health: The role of received stigma and perceived structural stigma
Source: Int J Soc Psychiatry. 2024 Nov 9;71(3):554–63. doi: 10.1177/00207640241296055 (PMC12012280; doi:10.1177/00207640241296055)
Supplement: sj-docx-1-isp-10.1177_00207640241296055 – Supplemental material for Testing the associations between poverty stigma and mental health: The role of received stigma and perceived structural stigma [file sj-docx-1-isp-10.1177_00207640241296055.docx]

Supplementary Materials

Supplementary Table 1: Bivariate analyses testing the associations between sociodemographic factors and poverty stigma.

Supplementary Table 2: Multivariate analyses testing the associations between sociodemographic factors and poverty stigma.

Supplementary Table 3: Bivariate analyses testing the associations between mental health outcomes, poverty stigma and sociodemographic factors.

Supplementary Table 1: Bivariate analyses testing the associations between sociodemographic factors and poverty stigma.

|  | Received stigma | | Perceived structural stigma | |
| --- | --- | --- | --- | --- |
|  | B (95% CI) | *β* | B (95% CI) | *β* |
| Age | -.05 (-.07 , -.03)*** | -.15 | -.04 (-.06 , -.02)*** | -.14 |
| Gender |  |  |  |  |
| Male / other gender identity (reference) |  |  |  |  |
| Female | .46 (-.11 , 1.04) | .05 | .80 (.20 , 1.39)** | .09 |
| Ethnicity |  |  |  |  |
| White (reference) |  |  |  |  |
| Other ethnicity | .37 (-.55 , 1.28) | .03 | -.89 (-1.84 , .05) | -.06 |
| Subjective social status | -.81 (-1.00 , -.63)*** | -.27 | -.87 (-1.06 , -.68)*** | -.28 |
| Financial hardship | .43 (.38 , .48)*** | .50 | .28 (.23 , .33)*** | .31 |
| Subjective poverty |  |  |  |  |
| No (reference) |  |  |  |  |
| Yes | 2.89 (2.29 , 3.49)*** | .29 | 2.22 (1.59 , 2.86)*** | .22 |
| Income |  |  |  |  |
| Below £10,000 (reference) |  |  |  |  |
| £10,000 - £15,999 | -.11 (-1.27 , 1.06) | -.01 | .36 (-.85 , 1.57) | .03 |
| £16,000 - £19,999 | -.60 (-1.85 , .65) | -.04 | -.29 (-1.59 , 1.01) | -.02 |
| £20,000 - £29,000 | -.91 (-1.96 , .13) | -.10 | .01 (-1.08 , 1.09) | .00 |
| £30,000 - £39,000 | -.95 (-2.17 , .28) | -.07 | -.02 (-1.30 , 1.26) | .00 |
| £40,000 - £49,000 | -2.16 (-3.77 , -.55)** | -.10 | .38 (-1.30 , 2.06) | .02 |
| £50,000 or more | -.74 (-2.28 , .81) | -.04 | .81 (-.80 , 2.43) | .04 |
| Education |  |  |  |  |
| Primary or secondary school (reference) |  |  |  |  |
| College or undergraduate degree | .26 (-.40 , .91) | .03 | .86 (.18 , 1.54)* | .09 |
| Postgraduate degree | .80 (-.14 , 1.74) | .06 | .30 (-.67 , 1.28) | .02 |
| Employment |  |  |  |  |
| Employed full-time (reference) |  |  |  |  |
| Employed part-time | -.18 (-.91 , .55) | -.02 | -.28 (-1.04 , .49) | -.03 |
| Out of work and looking for a job | .05 (-1.24 , 1.35) | .00 | .44 (-.92 , 1.80) | .02 |
| Out of work because of long-term sickness or disability | 2.45 (1.33 , 3.57)*** | .14 | 1.57 (.40 , 2.75)** | .09 |
| In education | -.07 (-1.52 , 1.38) | .00 | .21 (-1.31 , 1.73) | .01 |
| Looking after home or family | .50 (-.54 , 1.54) | .03 | -.10 (-1.19 , .99) | -.01 |
| Retired | -2.74 (-3.82 , -1.65)*** | -.16 | -2.35 (-3.49 , -1.22)*** | -.14 |
| Other employment status | -.26 (-1.68 , 1.16) | -.01 | -.74 (-2.23 , .75) | -.03 |
| Receives benefits |  |  |  |  |
| No (reference) |  |  |  |  |
| Yes | 1.82 (1.24 , 2.40)*** | .19 | 1.58 (.97 , 2.19)*** | .16 |
| Has used money/ debt advice in past 12 months |  |  |  |  |
| No (reference) |  |  |  |  |
| Yes | 2.75 (1.89 , 3.60)*** | .20 | 1.79 (.89 , 2.69)*** | .13 |
| Has used foodbank in past 12 months |  |  |  |  |
| No (reference) |  |  |  |  |
| Yes | 3.96 (2.98 , 4.94)*** | .25 | 1.69 (.64 , 2.73)** | .10 |
| Food security |  |  |  |  |
| High food security (reference) |  |  |  |  |
| Low food security | 2.94 (2.30 , 3.57)*** | .27 | 1.66 (.94 , 2.38)*** | .15 |
| Very low food security | 5.07 (4.48 , 5.65)*** | .51 | .3.48 (2.82 , 4.14)*** | .34 |

Note: B = unstandardised coefficient; CI = confidence interval; β = standardised coefficient

* p < .05, ** p < .01, *** p < .001

Supplementary Table 2: Multivariate analyses testing the associations between sociodemographic factors and poverty stigma.

|  | Received stigma | | Perceived structural stigma | |
| --- | --- | --- | --- | --- |
|  | B (95% CI) | *β* | B (95% CI) | *β* |
| Age | .00 (-.03 , .02) | -.01 | -.01 (-.04 , .01) | -.04 |
| Gender |  |  |  |  |
| Male / other gender identity (reference) |  |  |  |  |
| Female | -.02 (-.52 , .48) | .00 | .48 (-.09 , 1.05) | .05 |
| Ethnicity |  |  |  |  |
| White (reference) |  |  |  |  |
| Other ethnicity | .34 (-.46 , 1.14) | .02 | -1.15 (-2.06 , -.23)* | -.08 |
| Subjective social status | -.16 (-.35 , .03) | -.05 | -.62 (-.84 , -.39)*** | -.20 |
| Financial hardship | .26 (.18 , .33)*** | .30 | .09 (.01 , .18)* | .10 |
| Subjective poverty |  |  |  |  |
| No (reference) |  |  |  |  |
| Yes | -.70 (-1.42 , .02) | -.07 | -.07 (-.89 , .76) | -.01 |
| Income |  |  |  |  |
| Below £10,000 (reference) |  |  |  |  |
| £10,000 - £15,999 | .11 (-.89 , 1.10) | .01 | .67 (-.47 , 1.81) | .05 |
| £16,000 - £19,999 | .46 (-.63 , 1.55) | .03 | .71 (-.54 , 1.96) | .05 |
| £20,000 - £29,000 | .22 (-.74, 1.18) | .02 | 1.15 (.05 , 2.25)* | .12 |
| £30,000 - £39,000 | .23 (-.91 , 1.38) | .02 | 1.56 (.25 , 2.87)* | .11 |
| £40,000 - £49,000 | -.90 (-2.35, .54) | -.04 | 1.90 (.24 , 3.56)* | .09 |
| £50,000 or more | .55 (-.86 , 1.96) | .03 | 2.23 (.62 , 3.85)** | .11 |
| Education |  |  |  |  |
| Primary or secondary school (reference) |  |  |  |  |
| College or undergraduate degree | .43 (-.14 , .99) | .05 | 1.03 (.38 , 1.67)** | .11 |
| Postgraduate degree | .99 (.16 , 1.81)* | .08 | .66 (-.28 , 1.61) | .05 |
| Employment |  |  |  |  |
| Employed full-time (reference) |  |  |  |  |
| Employed part-time | -.09 (-.76 , .58) | -.01 | -.04 (-.81 , .73) | .00 |
| Out of work and looking for a job | -.33 (-1.52 , .86) | -.02 | .31 (-1.05 , 1.67) | .02 |
| Out of work because of long-term sickness or disability | 1.66 (.57 , 2.76)** | .10 | .80 (-.45 , 2.06) | .05 |
| In education | .35 (-.95 , 1.66) | .02 | .82 (-.67 , 2.31) | .04 |
| Looking after home or family | .32 (-.63 , 1.27) | .02 | -.32 (-1.41 , .77) | -.02 |
| Retired | -.44 (-1.55 , .66) | -.03 | -.10 (-1.36 , 1.16) | -.01 |
| Other employment status | -.28 (-1.54 , .98) | -.01 | -.58 (-2.02 , .87) | -.03 |
| Receives benefits |  |  |  |  |
| No (reference) |  |  |  |  |
| Yes | .12 (-.46 , .70) | .01 | .58 (-.08 , 1.25) | .06 |
| Has used money/ debt advice in past 12 months |  |  |  |  |
| No (reference) |  |  |  |  |
| Yes | .73 (-.04 , 1.49) | .05 | .36 (-.52 , 1.24) | .03 |
| Has used foodbank in past 12 months |  |  |  |  |
| No (reference) |  |  |  |  |
| Yes | 1.34 (.44 , 2.25)** | .08 | -.09 (-1.13 , .94) | -.01 |
| Food security |  |  |  |  |
| High food security (reference) |  |  |  |  |
| Low food security | 1.59 (.89 , 2.30)*** | .15 | .72 (-.09 , 1.53) | .06 |
| Very low food security | 2.59 (1.81 , 3.37)*** | .26 | 1.79 (.90 , 2.68)*** | .17 |

*Note:* B = unstandardised coefficient; CI = confidence interval; *β* = standardised coefficient

** p* < .05, ** *p* < .01, *** *p* < .001

Supplementary Table 3: Bivariate analyses testing the associations between mental health outcomes, poverty stigma and sociodemographic factors.

|  | Anxiety and depression (PHQ-4) | | Mental well-being (SWEMWBS) | |
| --- | --- | --- | --- | --- |
|  | B (95% CI) | *β* | B (95% CI) | *β* |
| Received stigma | .40 (.35 , .45)*** | .47 | -.29 (-.34 , -.24)*** | -.33 |
| Perceived structural stigma | .31 (.26 , .36)*** | .38 | -.26 (-.31 , -.21)*** | -.30 |
| Age | -.06 (-.08 , -.05)*** | -.23 | .06 (.04 , .08)*** | .23 |
| Gender |  |  |  |  |
| Male / other gender identity (reference) |  |  |  |  |
| Female | .74 (.26 , 1.22)** | .10 | -.33 (-.83 , .17) | -.04 |
| Ethnicity |  |  |  |  |
| White (reference) |  |  |  |  |
| Other ethnicity | .20 (-.58 , .97) | .02 | -.49 (-1.29 , .31) | -.04 |
| Subjective social status | -.88 (-1.03 , -.73)*** | -.35 | .83 (.67 , .98)*** | .32 |
| Financial hardship | .36 (.32 , .40)*** | .49 | -.32 (-.36 , -.28)*** | -.42 |
| Subjective poverty |  |  |  |  |
| No (reference) |  |  |  |  |
| Yes | 2.49 (1.99 , 3.00)*** | .30 | -2.46 (-2.98 , -1.93)*** | -.29 |
| Income |  |  |  |  |
| Below £10,000 (reference) |  |  |  |  |
| £10,000 - £15,999 | .06 (-.93 , 1.04) | .01 | -.16 (-1.17 , .85) | -.02 |
| £16,000 - £19,999 | -.81 (-1.87 , .25) | -.07 | .39 (-.70 , 1.48) | .03 |
| £20,000 - £29,000 | -.79 (-1.67 , .09) | -.10 | .89 (-.01 , 1.80) | .11 |
| £30,000 - £39,000 | -.79 (-1.83 , .25) | -.07 | .65 (-.42 , 1.72) | .06 |
| £40,000 - £49,000 | -.69 (-2.05 , .68) | -.04 | .41 (-.99 , 1.82) | .02 |
| £50,000 or more | -.63 (-1.94 , .68) | -.04 | .38 (-.97 , 1.73) | .02 |
| Education |  |  |  |  |
| Primary or secondary school (reference) |  |  |  |  |
| College or undergraduate degree | .30 (-.25 , .85) | .04 | .15 (-.42 , .72) | .02 |
| Postgraduate degree | .79 (-.01 , 1.58) | .07 | -.50 (-1.32 , .32) | -.04 |
| Employment |  |  |  |  |
| Employed full-time (reference) |  |  |  |  |
| Employed part-time | -.07 (-.68 , .54) | -.01 | .23 (-.40 , .86) | .03 |
| Out of work and looking for a job | .40 (-.69 , 1.48) | .02 | -1.12 (-2.24 , .01) | -.06 |
| Out of work because of long-term sickness or disability | 2.80 (1.86 , 3.73)*** | .19 | -1.51 (-2.48 , -.54)** | -.10 |
| In education | .25 (-.97 , 1.46) | .01 | .14 (-1.12 , 1.40) | .01 |
| Looking after home or family | -.20 (-1.07 , .67) | -.02 | .39 (-.51 , 1.29) | .03 |
| Retired | -2.37 (-3.28 , -1.46)*** | -.17 | 3.02 (2.08 , 3.96)*** | .21 |
| Other employment status | .39 (-.79 , 1.58) | .02 | -.69 (-1.92 , .54) | -.04 |
| Receives benefits |  |  |  |  |
| No (reference) |  |  |  |  |
| Yes | 1.18 (.68 , 1.67)*** | .15 | -.82 (-1.34 , -.31)*** | -.10 |
| Has used money/ debt advice in past 12 months |  |  |  |  |
| No (reference) |  |  |  |  |
| Yes | 1.86 (1.14 , 2.59)*** | .16 | -.99 (-1.75 , -.24)* | -.08 |
| Has used foodbank in past 12 months |  |  |  |  |
| No (reference) |  |  |  |  |
| Yes | 2.44 (1.59 , 3.28)*** | .18 | -1.76 (-2.63 , -.88)*** | -.13 |
| Food security |  |  |  |  |
| High food security (reference) |  |  |  |  |
| Low food security | 1.79 (1.24 , 2.34)*** | .20 | -1.62 (-2.21 , -1.03)*** | -.17 |
| Very low food security | 4.04 (3.54 , 4.55)*** | .48 | -3.53 (-4.07 , -2.99)*** | -.41 |
